# Statistical Properties of a Virtual Cohort for *In Silico* Trials Generated with a Statistical Anatomy Atlas

**DOI:** 10.1007/s10439-022-03050-8

**Published:** 2022-09-06

**Authors:** Antonino A. La Mattina, Fabio Baruffaldi, Mark Taylor, Marco Viceconti

**Affiliations:** 1 Department of Industrial Engineering, Alma Mater Studiorum - University of Bologna (IT), Bologna, Italy; 2 Medical Technology Lab, IRCCS Istituto Ortopedico Rizzoli, Via di Barbiano 1/10, 40136 Bologna, Italy; 3 Medical Device Research Institute, College of Science and Engineering, Flinders University, Adelaide, Australia

**Keywords:** Cohort expansion, *In silico* trials, Proximal femur fracture, Bone biomechanics, Statistical atlas

## Abstract

Osteoporosis-related hip fragility fractures are a catastrophic event for patient lives but are not frequently observed in prospective studies, and therefore phase III clinical trials using fractures as primary clinical endpoint require thousands of patients enrolled for several years to reach statistical significance. A novel answer to the large number of subjects needed to reach the desired evidence level is offered by *In Silico* Trials, that is, the simulation of a clinical trial on a large cohort of virtual patients, monitoring the biomarkers of interest. In this work we investigated if statistical aliasing from a custom anatomy atlas could be used to expand the patient cohort while retaining the original biomechanical characteristics. We used a pair-matched cohort of 94 post-menopausal women (at the time of the CT scan, 47 fractured and 47 not fractured) to create a statistical anatomy atlas through principal component analysis, and up-sampled the atlas in order to obtain over 1000 synthetic patient models. We applied the biomechanical computed tomography pipeline to the resulting virtual cohort and compared its fracture risk distribution with that of the original physical cohort. While the distribution of femoral strength values in the non-fractured sub-group was nearly identical to that of the original physical cohort, that of the fractured sub-group was lower than in the physical cohort. Nonetheless, by using the classification threshold used for the original population, the synthetic population was still divided into two parts of approximatively equal number.

## Introduction

Fragility fractures due to osteoporosis are a heavy burden for the public health system worldwide:^35^ about 9 million new fragility fractures are estimated every year, with 1.6 million located at the hip.^18^ Although hip fractures represent about 20% of the total fragility fractures, the related health cost is much higher, exceeding 50%.^10^ This is because hip fracture treatment requires expensive hospitalization, surgery, and rehabilitation; moreover, this traumatic event often catastrophically affects patient life quality, leading to permanent invalidity or even death within one year.^28^ One of the main osteoporosis risk factors is ageing, so that more and more people are being affected by this disease from year to year, due to the ageing population in most developed countries.

To test new drugs, we need phase III clinical trials with a very high level of evidence. Osteoporosis therapies aim to reduce the incidence of fragility fractures, and any phase III clinical trial that uses fractures as primary clinical endpoint involves thousands of patients and requires years of follow-up, in order to observe a sufficient number of fracture events.^6,7,9,29^ The alternative is to use surrogate endpoints that are good predictors of the primary endpoint but can be observed better and/or earlier. The most popular surrogate biomarker for the incidence of proximal femur fragility fractures is the areal bone mineral density (aBMD) as measured by dual-energy X-ray absorptiometry (DXA).^2,33,34^ In spite the fact that aBMD correlates with the incidence of fragile fractures, when used as a risk predictor its stratification accuracy is modest,^3,5,21,32^ with half of the hip fractures experienced by patients classified as non-osteoporotic. A more recent alternative as surrogate biomarker is Biomechanical Computed Tomography (BCT), as it is referred in some studies.^19,20^ BCT uses knowledge on continuum mechanics and 3D morpho-densitometry provided by quantitative CT (QCT) scans to inform a mechanistic computer model that predicts the patient’s bone biomechanical strength (primary determinant of the risk of fracture). BCT bone strength shows a better stratification accuracy than aBMD, and thus performs better as surrogate biomarker, requiring smaller cohorts for the clinical trials.^3,5,24^

But the fundamental problem remains: testing new osteoporosis drugs is too complex. One can use the fracture endpoint (and follow-up patients for at least 5 years), or use aBMD (and recruit many patients to achieve the necessary discriminant power, and require a DXA per control), or use BCT (that needs less patients than aBMD but requires a CT scan per control). Also, animal testing is unfortunately not accurate in predicting efficacy in humans for this class of drugs, so it is not unusual to have osteoporosis drugs that fail for efficacy in phase III clinical trials (e.g., Novartis’ SMC021 drug).^17^ One emerging option are *In Silico* Trials.^22,30^ The idea is to use surrogate endpoints provided by computer simulations, such as BCT, to simulate the effect of the treatment being tested vs placebo or another drug already in use (comparator) on a large cohort of virtual patients. In the case of osteoporosis, the generation of virtual cohorts involves the description of population variability of the femur 3D morpho-densitometry, as provided by QCT, with statistical anatomy atlases informed by a collection of QCT done on a smaller cohort of physical patients. To do so, a simple yet powerful technique is active shape and appearance modelling (ASAM), that leverages principal component analysis (PCA) for the extraction of (at least linearly) uncorrelated features from the starting original population.^1,11,13,27^ However, due to the complex relationship between the femoral 3D morpho-densitometry and femur biomechanical strength, it is not clear if the resulting virtual cohort will present a distribution of bone strength values similar to that of the physical cohort used to generate the statistical atlas.

The aim of this paper is to test if the distribution of BCT femoral strength in a virtual cohort generated with a statistical atlas is comparable to that of the physical cohort from which the atlas was built.

## Materials and methods

### Original Cohort

The starting original cohort was composed by proximal femur scans of 94 postmenopausal women (age 75 ± 9 years, range 55–91; height 158 ± 6 cm, range 145–173; weight 63.6 ± 13.4 kg, range 31–101; BMI 25.45 ± 4.87 kg/m^2^, range 14.40–36.43; proximal femur aBMD 0.800 ± 0.125 g/cm^2^, range 0.502–1.079; femur neck aBMD 0.630 ± 0.096 g/cm^2^, range 0.425–0.840); the cohort was pair-matched, so that for each fractured woman there was an analogous one with same age, height and weight, but not fractured. The data were collected in a retrospective study approved by the Sheffield Local Research Ethics Committee; the details of the primary study are provided in Ref. 36. The CT scans were performed at 120 kVp with tube current modulation (range 80–200 mA), a pixel size of 0.74 mm × 0.74 mm and a slice thickness of 0.625 mm, and were calibrated off-line by using the European Spine Phantom; details are reported in Refs. 24,36. A local reference system was defined, with the origin in femur head centre, X axis towards patient head, and Y axis towards greater trochanter; the proximal femur models were also fitted with an anatomy atlas in order to estimate knee rotation centre position, as reported in Ref. 24.

### PCA-Based Cohort Expansion

**Figure 1:**
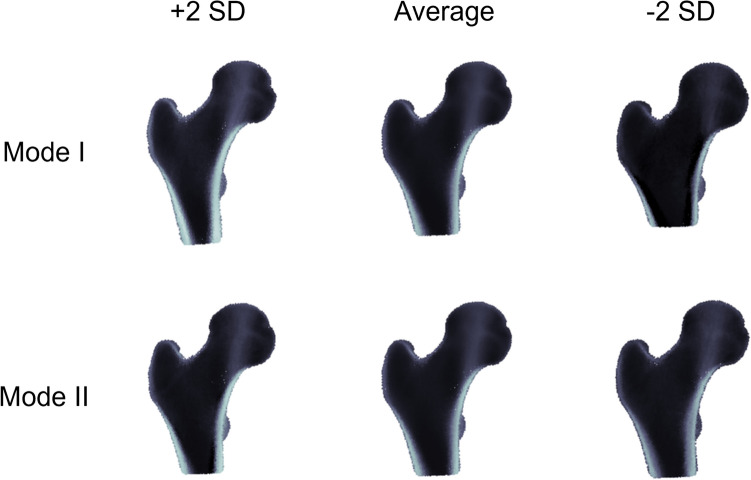
The first 2 modes of variation of the PCA-based statistical anatomy atlas.

A high-quality 10-nodes tetrahedral mesh (mean edge length 3 mm) of the femur (right side) with median dimensions was generated (ANSYS ICEM CFD 2019R2, ANSYS Inc., USA). The mesh (410,359 nodes + 4 landmarks (namely, femur head centre, patient head direction, greater trochanter direction, knee rotation centre), 295,589 elements) was subsequently morphed as described in Ref. 15 to fit the other 93 femurs (left femurs were reflected before the morphing operation), and the element-wise material properties were mapped (Bonemat V3)^26^ to obtain 94 isotopological patient-specific finite-element (FE) models. PCA was then applied to node and reference system point coordinates and to element Young moduli in order to obtain active shape and appearance models, respectively, which were then combined to create a single 1,526,678 × 93 matrix for the generation of synthetic models, as reported elsewhere.^27^ In Fig. 1 the first and second variation modes (together accounting for 41% of total variance) are represented, mainly consisting in scaling and slenderness of the proximal femur model together with general aBMD variation and cortical bone thickness. The PCA representation was not compact, and the first 70 components were needed to explain 95% of the total variance, similarly to what has been reported in literature for statistical appearance models of proximal femur;^8,12,25^ we used all the 93 principal components for the synthetic cohort generation. In order to generate principal component weights with the same original component distributions, we employed inverse transform sampling, a standard method used to generate random numbers with an arbitrary probability distribution, provided its inverse cumulative distribution function. Firstly, we calculated the empirical cumulative distribution function for each principal component, and approximated its inverse by piecewise linear interpolation. Then we generated uniformly distributed random numbers in the range 0.025–0.975 (equivalent to a ± 2 SD range for gaussian variables), and by applying the aforementioned inverse cumulative functions we converted them into random variables with the same distributions as the original principal components. We created 1080 synthetic femurs, of which 1044 were successfully used for the simulations, while the others showed excessive element distortion or convergence problems during some simulations. Cohort expansion was performed in MATLAB environment (MATLAB 2019b, MathWorks Inc., USA).

Patient heights and weights showed a weak correlation with femur neck aBMD and between themselves (Pearson’s correlation coefficient around 0.4), thus we assigned random weights and heights in dependence of femur neck aBMD, preserving the correlation between these parameters. Briefly, linear regression was applied to weights and aBMDs of the original population, according to the model

$$\mathrm{Weight}=a+b\times \mathrm{aBMD}+ \varepsilon$$

where $a$ is the intercept, $b$ is the slope, and $\varepsilon$ is a zero-mean random gaussian error with a certain variance. By using Pearson’s correlation definition, the error variance corresponding to a given correlation value can be calculated as

$$\mathrm{Var}\left(\varepsilon \right)=\mathrm{Var}\left(\mathrm{aBMD}\right)\times {b}^{2}\times \frac{1-{r}^{2}}{{r}^{2}}$$

where $\mathrm{Var}\left(X\right)$ is the variance of the stochastic variable $X$, and $r$ is the Pearson’s correlation coefficient between the two variables of interest. Similarly, heights were assigned with a bilinear regression with aBMD and weights.

#### FE Side-Fall Model

To evaluate femur strength, 28 non-linear simulations with different boundary conditions for each model were run, as described in Ref. 3 (ANSYS Mechanical APDL 2020R1, ANSYS Inc., USA). Briefly, at the centre of the femur head a 1000 N load was applied, spanning orientations from −30° to 30° in the antero-posterior direction, and 0° to 30° in medio-lateral direction (with steps of 10° in both directions). The distal femur diaphysis was allowed to rotate around knee rotation centre with a rotation axis perpendicular to the applied load direction, while a rigid plane was created in correspondence of the furthest femur node along load direction; non-linear frictionless contact between femur surface and the rigid plane was imposed as boundary condition.

#### Fracture Risk Estimators

The failure load for a particular angle side-fall was calculated as the load magnitude to apply in order to reach a principal tensile strain of 0.73% or a principal compressive strain of 1.04% (whichever was reached first) on the femur surface;^4^ the local strains were averaged over a sphere with 3 mm radius prior to fracture load estimation. Starting from the different angle failure loads, two fracture risk estimators were defined, namely, Minimum Side Fall (MSF) strength and Absolute Risk of Fracture at time 0 (ARF0). MSF was taken as the minimum of the failure loads on the different loading angles, while ARF0 calculation required the generation of a patient-specific distribution of side-fall load magnitudes, starting from patient height and weight; for details about the multiscale model used, see Ref. 5. Briefly, for each falling angle the fracture probability was calculated as the fraction of falling loads that exceeded the estimated critical load; the general fracture probability due to a side-fall (*P*) was then computed as the mean value of the double integral over the falling angles. Eventually, ARF0 was evaluated as the risk for the patient to experience a fracture due to at least one of the mutually exclusive falls in the following year, considering a rate of 0.65 fall per year:^5,14^ $\mathrm{ARF0}=1-{\left(1-P\right)}^{0.65}$.

Since in previous studies ARF0 was found to show a better stratification accuracy than MSF strength, hereinafter we will consider only ARF0.

### Statistical Analysis

Receiver Operating Characteristic (ROC) curve for the ARF0 fracture risk estimator was calculated for the physical cohort; the threshold for classification was chosen as that giving equal error rate. This optimal threshold was subsequently used also to classify the synthetic patients.

The distributions of ARF0 for both the physical cohort and the virtual cohort were tested for normality (Shapiro–Wilk, D’Agostino, and Anderson tests) and were found not gaussian (*p*-value < 0.05). Therefore, to test the distribution similarity a non-parametric test (Kolmogorov–Smirnov) was used. In addition, the ARF0 distributions were fitted with a two-component gaussian mixture model using a non-Bayesian expectation maximization algorithm, in order to separate contributions from fractured and non-fractured populations.

Fracture risk estimation and statistical analysis were performed using NumPy,^16^ SciPy,^31^ and Scikit-learn^23^ standard Python function libraries.

## Results

The distribution of ARF0 in the virtual cohort was found similar to that of the physical cohort, where it showed a bimodal distribution. A Gaussian Mixture model showed that the low-risk component of the distribution in the virtual cohort remained practically identical to that of the physical cohort (ARF0 = (28 ± 12) % for the virtual cohort, vs. ARF0 = (29 ± 12) % for the physical cohort). The high-risk component of the virtual cohort shifted towards higher fracture risk, with the average ARF0 rising from (55 ± 13) % for the physical cohort to (63 ± 15) % for the virtual cohort. However, the physical and the virtual ARF0 populations were found not statistically different (*p*-value > 0.05).

**Figure 2:**
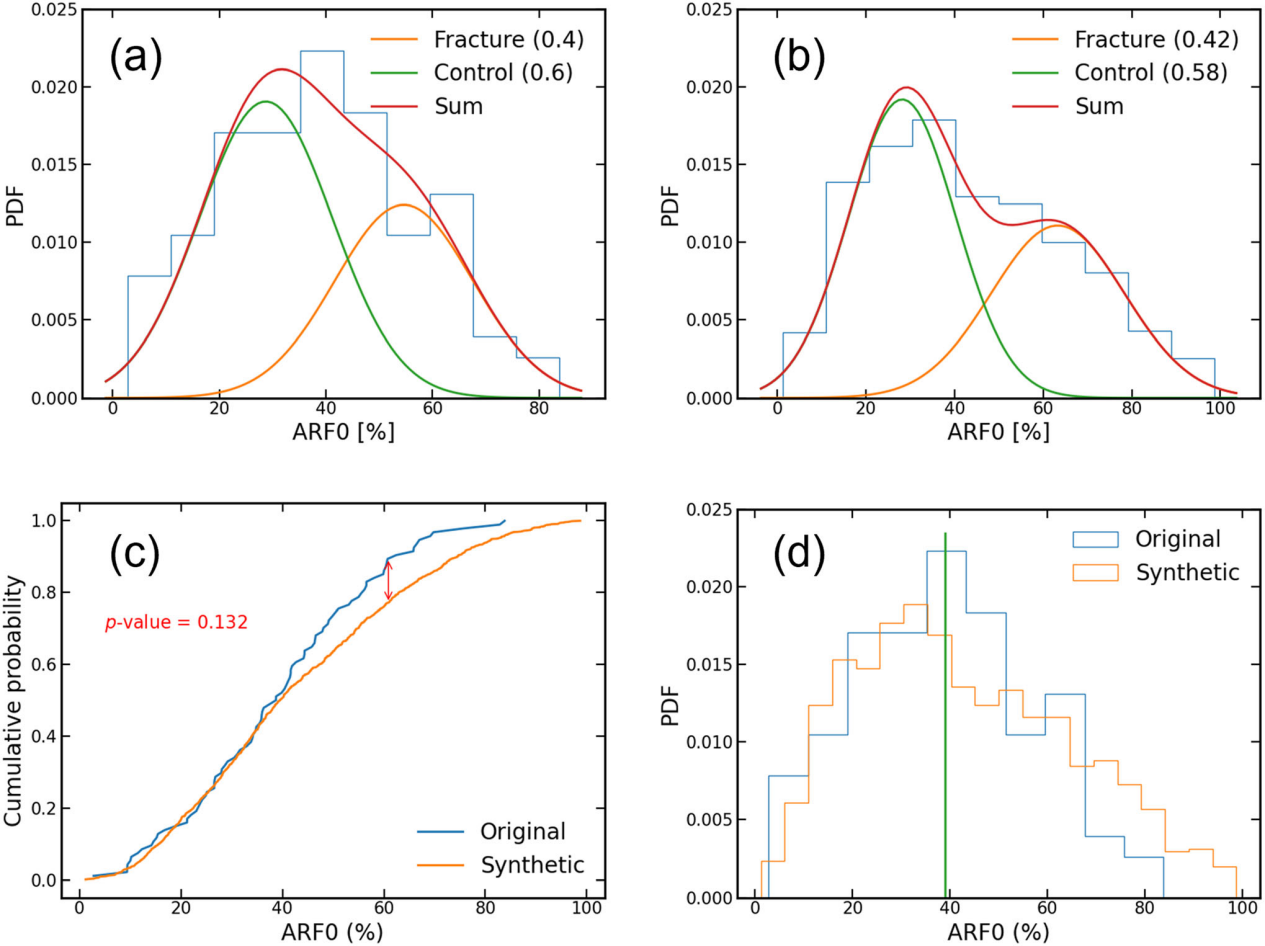
ARF0 distributions: ARF0 probability density functions for the original (a) and the synthetic (b) cohorts; (c) ARF0 cumulative probabilities of the cohorts and Kolmogorov–Smirnov test; (d) ARF0 distributions showing the threshold used for stratification.

The optimal threshold for ARF0 for the physical cohort was 39%. By using this threshold to classify the virtual subjects we found a stratification for fracture in the virtual cohort similar to that of the physical cohort, with 531 (50.9%) virtual subjects classified as fractured (Fig. 2).

Distribution of T-scores, heights, weights, and BMIs were not statistically different for the original and the virtual populations (*p*-value > 0.05).

## Discussion

The aim of this paper was to test if the distribution of BCT femoral strength in a virtual cohort generated with a statistical atlas was comparable to that of the physical cohort from which the atlas was built.

The statistical distribution of ARF0 in the virtual cohort presented the same bimodal shape observed in the physical cohort. When a Gaussian Mixture model was used to separate the two modes, we found that the distribution of the low-risk mode (typically associated to non-fractured subjects) was almost identical to that of the physical cohort. However, when we compared the distributions of the high-risk mode (typically associated to fractured subjects), we found a significant increase in the average value of ARF0, which moved from 55% in the physical cohort to 63% in the virtual cohort.

This result is not unexpected. A subject with no osteoporosis would have a T-score in the range + 1 to − 1, *e.g.*, within one standard deviation from the normal reference population. If we exclude subjects with diseases like osteopetrosis that increase bone density to pathological levels, it is unlikely that anyone can have a T-score greater than + 1. On the contrary, whereas we define osteoporotic a subject with T-score ≤ 2.5, it is not impossible to find subjects with T-score ≤ 5. In other words, physiology limits the upper boundary of the distribution, and thus oversampling does not change the distribution; pathology has no lower limit, and thus any oversampling will generate some cases with more severe osteoporosis and higher fracture risk. Also, the values of ARF0 can vary from 0 to 100%, and while the lower limit was well represented in the original cohort, its maximum ARF0 value was around 80%; on the other hand, the virtual cohort explored the entire range of possible ARF0 values.

But considering these virtual cohorts are used to predict who fractures and who does not, this difference in the high-risk mode distribution has a small impact. In fact, the threshold found optimal for the physical cohort stratified for fracture the virtual cohort in proportion (50.9%) quite close to that of the physical cohort (48.9%). Also, the distributions of atlas-derived biomarkers (T-score, femoral length, and BMI) were found to be very close to those measured in the physical cohort.

The main limit of this study is that the physical cohort we used to generate the virtual cohort is not the result of an observational trial, which would likely result into a mono-modal distribution of ARF0, but a carefully build pair-matched cohort, where for each post-menopausal woman that was admitted with a femoral neck fracture another osteopenic post-menopausal woman with same age, height, and weight who had no reported fractures was included. This creates the bi-modal distribution we described in both the physical cohort and in the virtual cohort derived from it. However, we see this as an advantage rather than a limitation. In other phase III clinical studies (for example,﻿ see Ref. 9) the inclusion–exclusion criteria were crafted to build a high-risk cohort and a low-risk cohort, to see the relative efficacy of the drug being tested on these two typical sub-populations. Using a Gaussian Mixture model, we are able to separate our 1000-subjects virtual cohort in two sub-cohorts of roughly 500 virtual subjects each, one at high risk and the other at low risk, similarly to the cited clinical studies.

In conclusions, statistical anatomy atlases informed by smaller physical cohorts can be used to generate much larger virtual cohorts that retain the essential statistical properties of the physical cohort. Thus, this approach can be used for cohort expansion in *In Silico* Trials, for example by converting 100 patient-specific models obtained in the phase II clinical trial into a 1000-subjects virtual cohort, with a level of evidence comparable to a phase III clinical trial.

## Data Availability

Model data are available in the University of Bologna institutional repository under the terms of CC BY-NC-SA 4.0 International license at the link http://doi.org/10.6092/unibo/amsacta/6891
